# Label-Free Nanostructured Biosensing Platform Based on Depolarized Dynamic Light Scattering for Rapid and Portable Detection of Immunoglobulins in Complex Biological Samples

**DOI:** 10.1021/acsomega.5c12965

**Published:** 2026-05-21

**Authors:** Caroline Magalh̃aes Junqueira, Kennedy Batista Goņcalves, Ĺıvia de Brito Macedo, Patrick Gonçalves Mendez, Nick Rocha Costa, Iara Borges Apolińario, Enzo Morrison Figueiredo Costa Jorge, Amanda Bonoto Goņcalves, Anna Carolina Pinheiro Lage, Daniel de Assis Santos, Fĺavio Guimar̃aes da Fonseca, Rosimeire Coura Barcelos, Ary Corr̂ea Junior, Luiz Orlando Ladeira, Oscar Nassif de Mesquita, Ĺıvia Siman Gomes

**Affiliations:** † Department of Microbiology, Federal University of Minas Gerais (UFMG), Belo Horizonte 31270-901, Minas Gerais, Brazil; ‡ Center of Technology in Nanomaterials and Graphene (CTNano), Federal University of Minas Gerais (UFMG), Belo Horizonte 31270-901, Minas Gerais, Brazil; § Department of Physics, Federal University of Minas Gerais (UFMG), Belo Horizonte 31270-901, Minas Gerais, Brazil; ∥ Rene Rachou Institute (Fiocruz Minas), Belo Horizonte 30190-002, Minas Gerais, Brazil; ⊥ GAD − Organic Chemistry Group, Federal University of São João del-Rei, Divińopolis 35501-296, Minas Gerais, Brazil

## Abstract

The dynamic behavior of nanoparticles in solution provides valuable insights for biomolecular detection. When nanoparticles exhibit optical anisotropy, their Brownian rotational diffusion can be monitored through depolarized dynamic light scattering (DDLS). For anisotropic particles such as gold nanorods (GNRs), the rotational diffusion coefficient scales inversely with the cube of their hydrodynamic radius, making DDLS highly sensitive to nanoscale binding events. Here, we report a label-free nanostructured biosensing platform based on DDLS that enables rapid, sensitive, and portable detection of immunoglobulins in complex samples. The system integrates bioconjugated GNRs with a custom-built, miniaturized DDLS device to achieve real-time, in loco measurements within approximately ≈10 s, without extensive sample preprocessing. As a proof-of-concept, GNRs functionalized with SARS-CoV-2 nucleocapsid proteins were used to detect specific immunoglobulins in clinical samples. The nanobiosensor exhibited high analytical sensitivity, with a detection limit of 0.16 ng/mL, as determined using spiked samples containing commercial monoclonal anti-N antibodies, highlighting its potential for rapid point-of-care immunoassays in complex biological environments.

## Graphical abstract

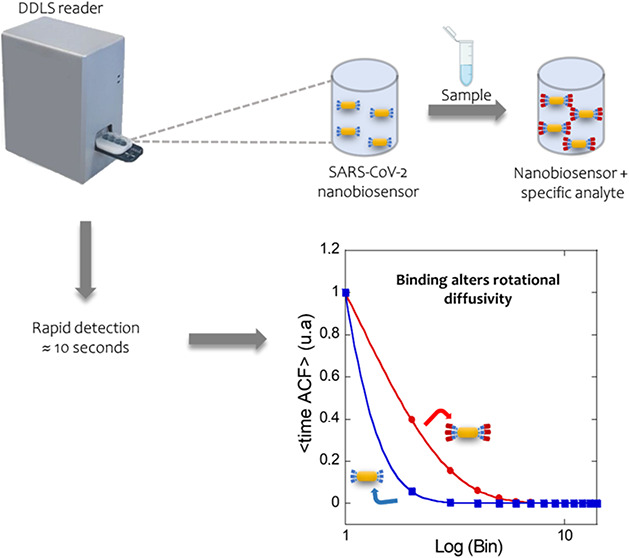

## Introduction

Despite continuous technological advances in biochemical sensing, rapid, simple, and sensitive detection of diverse biomolecular targets in complex fluid matrices remains a significant challenge.^1^ Among the strategies, most actively explored are label-free biosensors, devices capable of directly converting biomolecular recognition events into quantifiable physical signals.^2^ These biosensors offer potential advantages in terms of simplicity, speed, and the ability to monitor binding kinetics in real time. Gold nanoparticles (NPs) have been widely used as optical transducers in the development of biosensors for the direct detection of biomolecular interactions.^3–9^ These systems commonly exploit the phenomenon of surface plasmon resonance (SPR), a property of noble metal nanoparticles in which their interaction with light leads to two key effects: (i) a strong enhancement of electric fields near the nanoparticle surface, and (ii) characteristic peaks in the particle’s optical extinction spectrum. Resonance peaks are sensitive to changes in size, shape, and local refractive index, which is the basis of many sensing mechanisms.^3–5,10^

Brownian motion, which is the random movement of particles suspended in a fluid due to collisions with solvent molecules, can occur in two modes: translational and rotational. These movements are characterized by the translational (*D*_T_) and rotational (*D*_R_) diffusion coefficients, respectively. For anisotropic particles, such as nanorods, translational and rotational motions are distinguishable. Although *D*_T_ is inversely proportional to the hydrodynamic radius (described by the Stokes–Einstein equation), *D*_R_ scales with the inverse cube of the radius, making it highly sensitive to small changes in particle size and therefore a valuable parameter for biosensing applications. Dynamic light scattering (DLS) is a widely used, noninvasive technique for characterizing the Brownian motion of nanoscale and microscale particles suspended in fluid. From the diffusion coefficients measured by DLS, valuable information can be inferred such as the hydrodynamic size of the particles, particle interactions, and aggregation states. A conventional DLS setup comprises a laser source, optical components, a photodetector, a digital autocorrelator, and a computer. Briefly, the laser beam impinges on the colloidal suspension, and the Brownian motion of the particles causes temporal fluctuations in the local dielectric environment, leading to scattering of light at various angles. Depending on their phase, scattered light waves can interfere constructively or destructively, producing an intensity signal that fluctuates over time.^11,12^ This signal comprises a polarized component (VV) and a depolarized component (VH), with the latter arising only when the scatters are optically anisotropic. A polarizer placed before the detector selects the component of the scattered signal, and a digital autocorrelator computes the time autocorrelation functions (time-ACF) of the fluctuations of the scattered light intensity. For a colloidal suspension of monodisperse particles, the time-ACF decays exponentially with an exponential time constant (τ*t*) related to the diffusion coefficient of the scatterers.

DLS is a powerful tool for characterizing NP aggregation dynamics and has been successfully applied to the study of suspensions of bioconjugated spherical gold nanoparticles (sNPs) to monitor their molecular interactions with proteins,^6^ nucleic acids,^8,13^ small molecules,^7^ and microorganisms.^14^ This strategy, commonly referred to as DLS-based biosensing, is based on detecting variations in the aggregation state of sNPs induced upon analyte binding. Despite its broad application and high sensitivity,^15,16^ this methodology presents notable limitations, as NPs are highly susceptible to aggregation under fluctuations in pH, ionic strength, or temperature, which compromises the measurement reliability. Moreover, the associated colorimetric detection can be inconsistent when applied to intrinsically colored or turbid samples.

The present study employs measurements of the rotational diffusion coefficient using the depolarized dynamic light scattering (DDLS) technique for biosensing applications, utilizing a colloidal dispersion of gold nanorods (GNRs) as anisotropic optical transducers.^14,17,18^ Gold nanorods have cylindrical elongated shapes and are also extensively used in the field of biosensing. The technique has been previously employed for the monitoring of aggregation of functionalized nanoparticles for biosensing applications.^16,19–23^ The proposed methodology presents an enhanced sensitivity—due to the cubic behavior of *D*_R_ with respect to the length of the particles — and higher specificity than traditional DLS-based sensors, since they do not depend on the aggregation state of the particles.^20^

DDLS signals are usually very weak and laborious to measure, and this limitation is contoured here using GNRs with maximized scattering efficiency and a camera for the detection of the scattered light.^24–27^ Figure 1 illustrates the design of our DDLS-based biosensor: (A) GNRs are bioconjugated at their ends with molecular recognition elements (e.g., proteins, nucleic acids, or small molecules) providing nanobiosensors (NB) and (B) the sensing mechanism, which is based on alterations on rotational diffusion coefficient, Δ*D*_R_, of the NBs upon analyte binding. Δ*D*_R_ is given by ⟨*D*_RS_⟩ – ⟨*D*_R0_⟩, where *D*_R0_ is related to the rotational diffusion coefficients of the bare nanobiosensors in solution and *D*_RS_ is related to the rotational diffusion coefficients of nanobiosensors after sample addition. The time-ACFs are automatically calculated and analyzed by a portable DDLS apparatus, which returns the exponential time constant values (*τ*) or *D*_R_^–1^. The biosensing methodology is employed here to detect IgG antibodies against the nucleocapsid protein of SARS-CoV-2, produced in response to COVID-19 infection. Artificial and clinical samples are used to investigate and validate the proposed technology.

**Figure 1:**
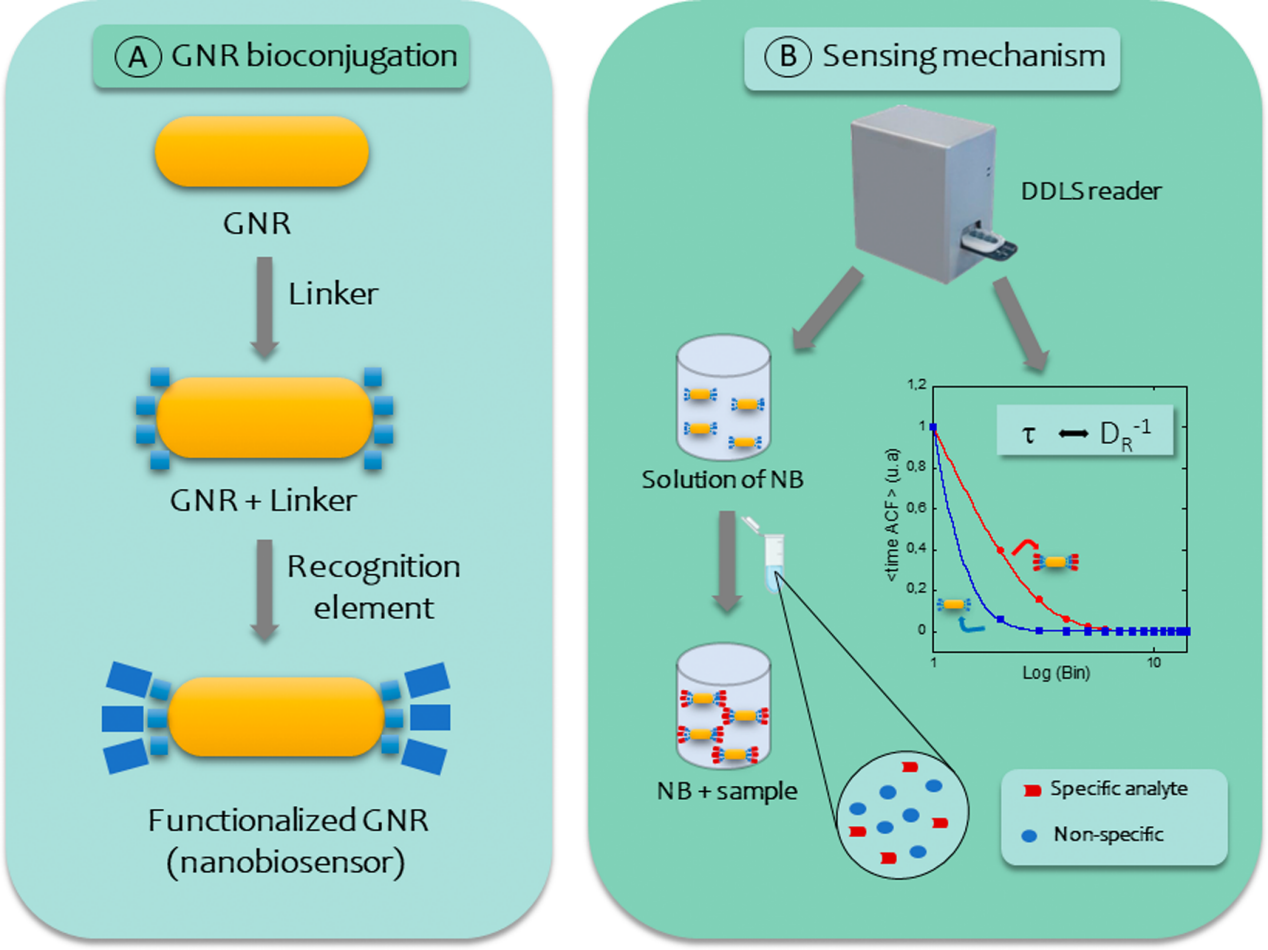
DDLS-based biosensor methodology. (A) Bioconjugation of GNRs. First, GNRs are synthesized with a specific surface plasmon resonance peak and are modified with a linker that binds to their end surfaces. Recognition elements bind onto the functionalized surfaces, forming nanobiosensors (NB). (B) Biosensing mechanism: a serum sample containing analytes is added to the dispersion of NBs, and their interaction induces changes in the rotational diffusivity. *D*_R_^–1^ values are directly obtained through the analysis of time-ACFs measured by the DDLS reader.

## Experimental Section

### Bioconjugated GNR

The gold nanorods (GNRs) used in this study exhibited an aspect ratio (AR = length/diameter) of approximately 2.2, corresponding to a longitudinal surface plasmon resonance (LSPR) peak near 650 nm. All nanoparticles were synthesized using a seedless growth method adapted from Wang et al.^28^ and characterized by UV–Vis spectroscopy (Thermo Scientific, Varioskan LUX), ζ-potential measurements (Litesizer 500, Anton Paar, Austria), transmission electron microscopy (Tecnai G2–20 SuperTwin 200 kV, Thermo Fisher/FEI), and Depolarized Dynamic Light Scattering (DDLS). After synthesis, GNRs with approximately 2.2 of AR were functionalized with 11– mercaptoundecanoic acid (MUA, 95%; Sigma-Aldrich, St. Louis, MO, USA; Cat. No. 450561) to enable covalent binding of the SARS-CoV-2 nucleocapsid (N) protein,^29^ as described by Yu and Irudayaraj^30^ Details regarding recombinant N-protein production are provided in Supporting Data S1.

Typically, cetyltrimethylammonium bromide (CTAB) molecules are preferentially adsorbed on the {110} and {100} facets of GNRs, forming a stable bilayer, while adsorption on the {111} end facets are comparatively weaker.^31,32^ This crystallographic anisotropy results in the exposure of the nanorod ends/caps, which exhibit higher affinity for thiolated ligands. Consequently, 11-mercaptoundecanoic acid (MUA) molecules preferentially bind to gold nanorod caps, rather than promoting a homogeneous ligand exchange over the entire surface. This strategy preserves CTAB on GNRs lateral facets, thereby maintaining colloidal stability. For this purpose, 1 mL of a 5 mM MUA ethanol solution was added to 50 mL of a 200 pM GNR suspension containing 10 mM CTAB (99%; Sigma-Aldrich; Cat. No. H6269) and stirred gently for 24 h at room temperature. The resulting GNR + MUA conjugates were collected by centrifugation at 10,000*g* for 10 min and redispersed in 10 mM CTAB to a final concentration of 200 pM. 10 mL of GNR + MUA suspension at 200 pM was activated by adding freshly prepared 0.4 M *N*-(3-dimethylaminopropyl)-*N*-ethylcarbodiimide hydrochloride (EDC–HCl, 99%; Sigma-Aldrich; Cat. No. 03449) and 0.1 M *N*-hydroxysuccinimide (NHS, 98%; Sigma-Aldrich; Cat. No. 130672), followed by sonication for 30 min at 4 °C. The samples were collected by centrifugation at 5000*g* for 10 min and resuspended in 10 mL of buffer containing 3 mM CTAB, 10 mM Tris base (Cytiva 17-1321-01) and 3 μM ethylenediaminetetraacetic acid disodium salt dihydrate (EDTA–Na2, ACS reagent, 99–101%; Sigma-Aldrich; Cat. No. E4884), pH 7.8.

To perform bioconjugation, 6 μL of a 21 mM SARS-CoV-2 N-protein solution was added to activated GNRs and incubated under gentle mixing for 12 h at room temperature. The resulting functionalized nanorods—hereafter referred to as nanobiosensors (NB)—were purified by five consecutive centrifugation cycles (2000*g*, 5 min each) and resuspended in storage buffer (1 mM CTAB, 10 mM Tris, 0.1% ProClin, 3 μM EDTA, pH 7.4) at a final concentration of 150 pM. Control experiments were carried out using nonactivated GNRs under identical conditions.

### Samples

Artificial samples were prepared by spiking a human-serum-based matrix with defined concentrations of monoclonal antibodies (mAb) against the SARS-CoV-2 nucleocapsid (N) protein (clone 6H3-G1; Cat. #MABF2787, Merck Millipore, Burlington, MA, USA). The antibody used corresponds to an antibody fragment (≈53 kDa), which recognizes an epitope within the N protein. Despite this reduced molecular size compared to full-length IgG (≈150 kDa), the fragment contributed to measurable changes in the rotational diffusion coefficient. A human-based serum matrix, free of target analytes, was used as the biological matrix to reproduce the native serum environment and to provide a consistent background for evaluating the binding behavior of the functionalized gold nanorods (GNRs).

Clinical serum samples were provided by the CTVacinas Center and previously characterized for SARS-CoV-2 antibodies by ELISA and RT-qPCR, as described in Table S1 (Supporting Information S2). The use of patient and control sera was approved by the UFMG Ethics Committee and the National Research Ethics Committee (CAAE: 1686320.0.0000.5149).

All samples (serum-based matrix and clinical serum) were diluted 1:1000 in phosphate-buffered saline (PBS; pH 7.4; Sigma-Aldrich D8537, St. Louis, MO, USA) and underwent further dilution during the assay. More precisely, controlled volumes of the diluted serum were added to the nanobiosensor suspension (350 μL at 130 pM) and gently homogenized by pipetting. Measurements were carried out at 25 °C using 96-well untreated flat-bottom microplates (Corning Inc., USA).

### DDLS Measurements

The custom-built DDLS apparatus (Figure 2A)^33^ featured a simplified configuration relative to conventional setups. It consisted of a 650 nm, 40 mW laser diode (Laserline) with elliptical beam geometry and a spot size of approximately 1 mm at the sample plane; a polarizing beam splitter cube (Thorlabs); a collimating lens (Thorlabs) for beam focusing and backscattered light collection; and a converging lens (Thorlabs) to project the scattered light onto a CMOS sensor (Ivision Co.). In this design, the sensor pixels act as an array of photodetectors that collect light at nearly identical scattering angles, enabling the simultaneous acquisition of multiple DDLS measurements from a single capture and ensuring a high signal-to-noise ratio. The device operated at a fixed scattering angle of 173°, which improves the detection of light scattered by small particles rather than aggregates, corresponding to a scattering vector of *q* = 2.57 × 10^7^ m^–1^ (*n* = 1.33). The time-ACFs are computed and fitted directly on the camera board, and the resulting exponential time constant values τ are displayed in real time through dedicated software on a PC or portable device.

**Figure 2:**
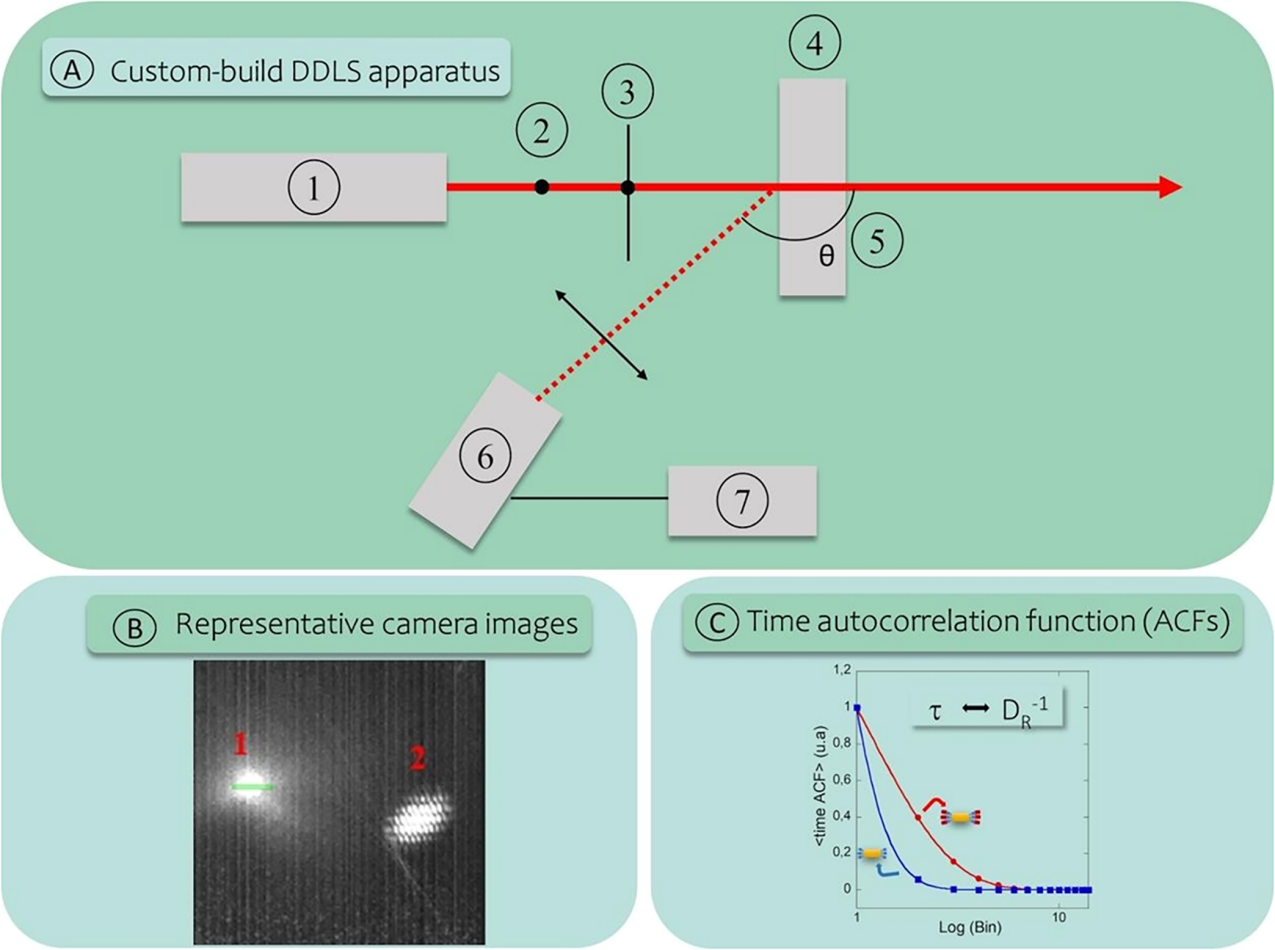
Schematic representation and operation of the custom-built DDLS setup. (A) Optical layout of the system, comprising: (1) laser diode; (2) polarized beam splitter cube; (3) collimating lens; (4) sample cell; (5) fixed scattering angle (θ); (6) CMOS sensor; (7) computer for data acquisition. (B) Representative image captured by the camera, where spot (1) corresponds to the dynamic scattering signal and spot (2) to the static laser reflection. The green area represents the region of interest (ROI), defined as two rows of 64 pixels each. (C) Time autocorrelation functions (ACFs) obtained from DDLS measurements. The decay of the autocorrelation curve reflects variations in the rotational diffusion coefficient induced by nanobiosensor–analyte interactions.

Each DDLS measurement comprised 1000 frames of 8-bit, 64-pixel images (ROI shown in Figure 2B) acquired at a rate of 42,533 Hz. Normalized intensity time-ACFs were calculated for each pixel and subsequently ensemble-averaged to enhance statistical accuracy. In the proposed configuration, the translational diffusion component can be neglected, and the time-ACF exponential time constant (τ) is equal to *D*_R_^–1^. The corresponding equations are provided in Supporting Data S3–S5. The acquisition and processing of each data set took approximately 10 s, allowing a rapid determination of *D*_R_^–1^.

The time-dependent intensity fluctuations of depolarized scattered light were analyzed using the time autocorrelation function obtained from DDLS measurements. The resulting autocorrelation curves exhibited characteristic exponential decay, whose exponential time constant (τ) is directly related to the inverse of the rotational diffusion coefficient (*D*_R_^–1^) of the anisotropic particles. By fitting the experimental data to a single exponential decay model, the value *D*_R_^–1^ was extracted. The use of a single exponential fitting model is justified from an experimental standpoint. Measurements are performed using a camera with a high acquisition rate, such that the detection window for a complete ACF is approximately 23 ms.

Under these conditions, the ACFs obtained for gold nanorods, the nanobiosensor, and the nanobiosensor in the presence of the sample exhibit well-behaved decays that can be adequately described by a single exponential, with low fitting residuals (Figures S1 and S2). Representative ACF curves (Figure 2C) illustrate that faster decay rates correspond to higher rotational mobility, consistent with smaller nanoparticles (e.g., the free nanobiosensor, represented by the blue curve), whereas slower decay rates indicate lower rotational mobility, associated with larger particles resulting from nanobiosensor binding to the specific analyte (represented by the red curve).

## Results and Discussion

### Characterization of GNRs and Nanobiosensors

Transmission electron microscopy (TEM) images of the synthesized GNRs (Figure 3A) confirmed a uniform cylindrical morphology with minimal occurrence of amorphous or irregular structures. Geometric analysis of 161 particles revealed an average length of (47 ± 8) nm and a diameter of (22 ± 4) nm, which yields an aspect ratio as described in Table S2 (Supporting Information).

**Figure 3:**
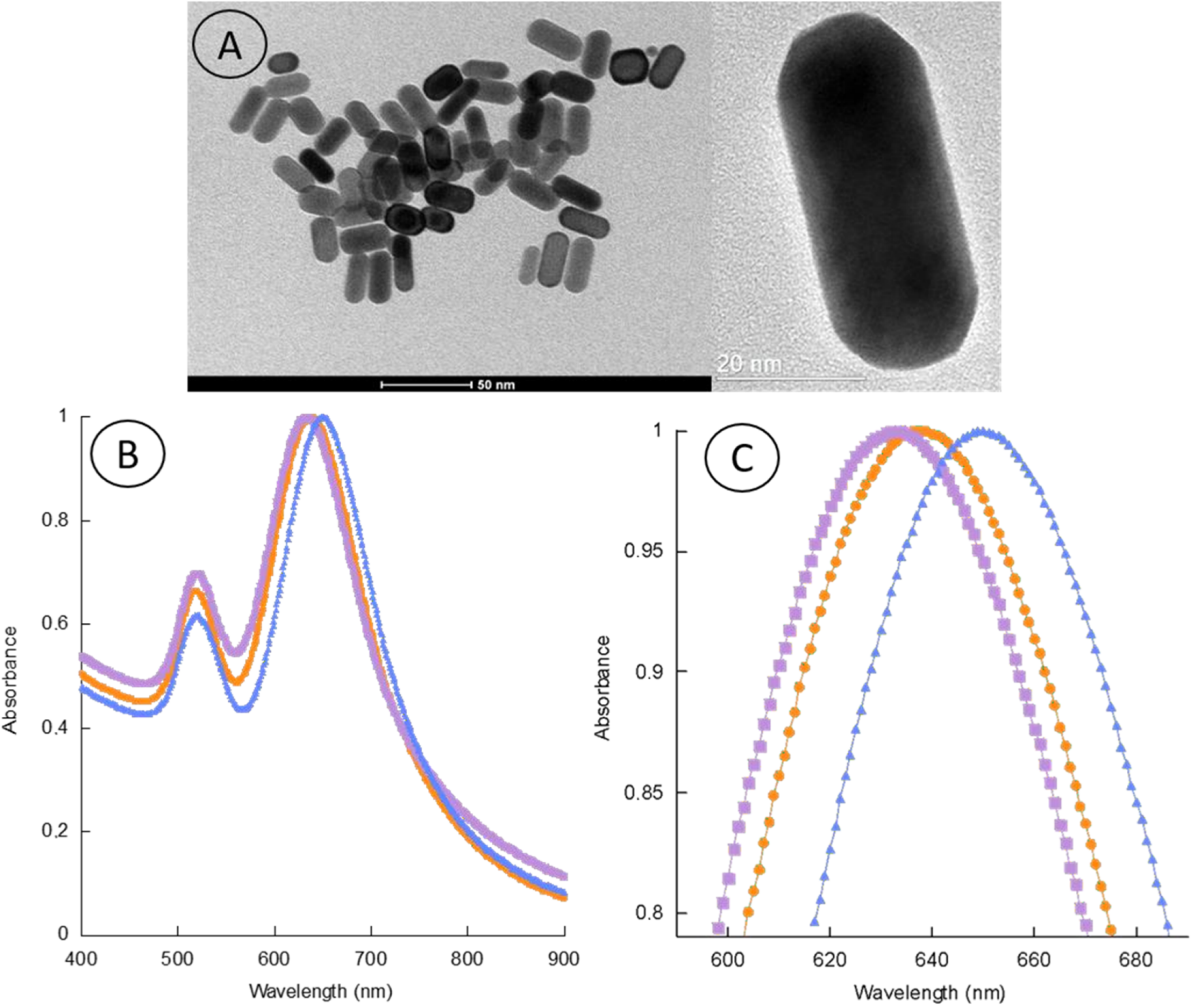
(A) TEM image of GNRs showing their anisotropic morphology and uniform size distribution. (B) UV–Vis absorption spectra of AR = 2.2 GNRs (blue triangles), GNR + MUA (orange dots), and assembled biosensors (purple squares). (C) Enlarged view of panel (B) highlighting the wavelength shifts associated with nanobiosensor construction.

The UV–Vis absorption spectra (Figure 3B, blue triangles) exhibited distinct longitudinal and transverse surface plasmon resonance peaks (LSPR and TSPR) centered at (650 ± 2) nm and (520 ± 3) nm, respectively. Upon MUA functionalization (orange dots) and subsequent nanobiosensor assembly (purple squares), the longitudinal peak experienced blue shifts of 6 and 9 nm, respectively. This effect can be understood considering that MUA binds preferentially to the nanorod ends, regions of high curvature where the collective oscillation of electrons is most intense (hot spot region). The localized functionalization in these regions alters the surface charge distribution and increases the restoring force acting on the free electrons during the plasmonic oscillation, leading to a higher oscillation frequency and, consequently, a shift of the resonance to shorter wavelengths (blue shift). After functionalization with the N protein, an additional blue shift is observed, indicating that the bioconjugation further modifies the electronic environment at the nanorod tips. The presence of the protein reinforces charge redistribution and alters the local electromagnetic coupling at the hot spots, further increasing the effective frequency of the longitudinal plasmon mode (Figure 3C).^34^ The absence of peak broadening further suggests that no aggregation occurred during functionalization or bioconjugation, consistent with ζ-potential data. Dynamic depolarized light scattering (DDLS) measurements yielded *D*_R_^–1^ values of (12 ± 1), (12 ± 2), and (17 ± 2) μs for GNR, GNR + MUA, and nanobiosensors (NB), respectively. The effective increase observed for nanobiosensors corresponds to an estimated elongation of *L* = (5 ± 1) nm, as described in eq S10 in the Supporting Information, at each tip of the nanorod, consistent with the theoretical globular size of the SARS-CoV-2 N protein (≈48 kDa).^35^

The ζ-potential measurements showed a surface potential of (+55 ± 1) mV for the as-synthesized GNRs, reflecting a strong electrostatic stabilization in suspension. Following MUA modification, the potential decreased slightly to (+50 ± 1) mV, and further to (+46 ± 2) mV after bioconjugation. This systematic reduction also indicates partial surface functionalization and charge screening induced by molecular attachment. All measurements were performed in triplicate, and additional characterization data are provided in Supporting Data S5.

### Evaluation of the Nanobiosensor’s Analytical Sensitivity and Selectivity Using Monoclonal Antibodies (mAbs)

The analytical sensitivity and selectivity of the DDLS-based biosensor were evaluated by using monoclonal antibodies (mAbs) specific to the SARS-CoV-2 nucleocapsid (N) protein. Artificially positive samples were prepared by spiking a human-serum-based matrix with known concentrations of mAbs. Wells containing nanobiosensors at a concentration of 130 pM were titrated with serum matrix volumes containing mAb at concentrations ranging from 0.6 pM (0.03 ng/mL) to 800 pM (43.2 ng/mL), hereafter termed the positive condition. The serum matrix alone was used as the negative control to evaluate nonspecific background responses.

After each incremental volume addition, the system was homogenized by gentle pipetting for 2 min. Subsequently, 60 ACFs measurements were collected over a period of 5 min, and the average value of ⟨*D*_R_^–1^⟩ was obtained. ⟨*D*_R_^–1^⟩ was then subtracted from the initial baseline for the nanobiosensor, *D*_R_^–1^ = (17 ± 2) μs, and for control, *D*_R_^–1^ = (12 ± 2) μs to produce ⟨Δ*D*_R_^–1^⟩ points, as presented in Figure 4. The complete kinetic data set is shown in Figure S2. All measurements were performed in triplicate.

**Figure 4:**
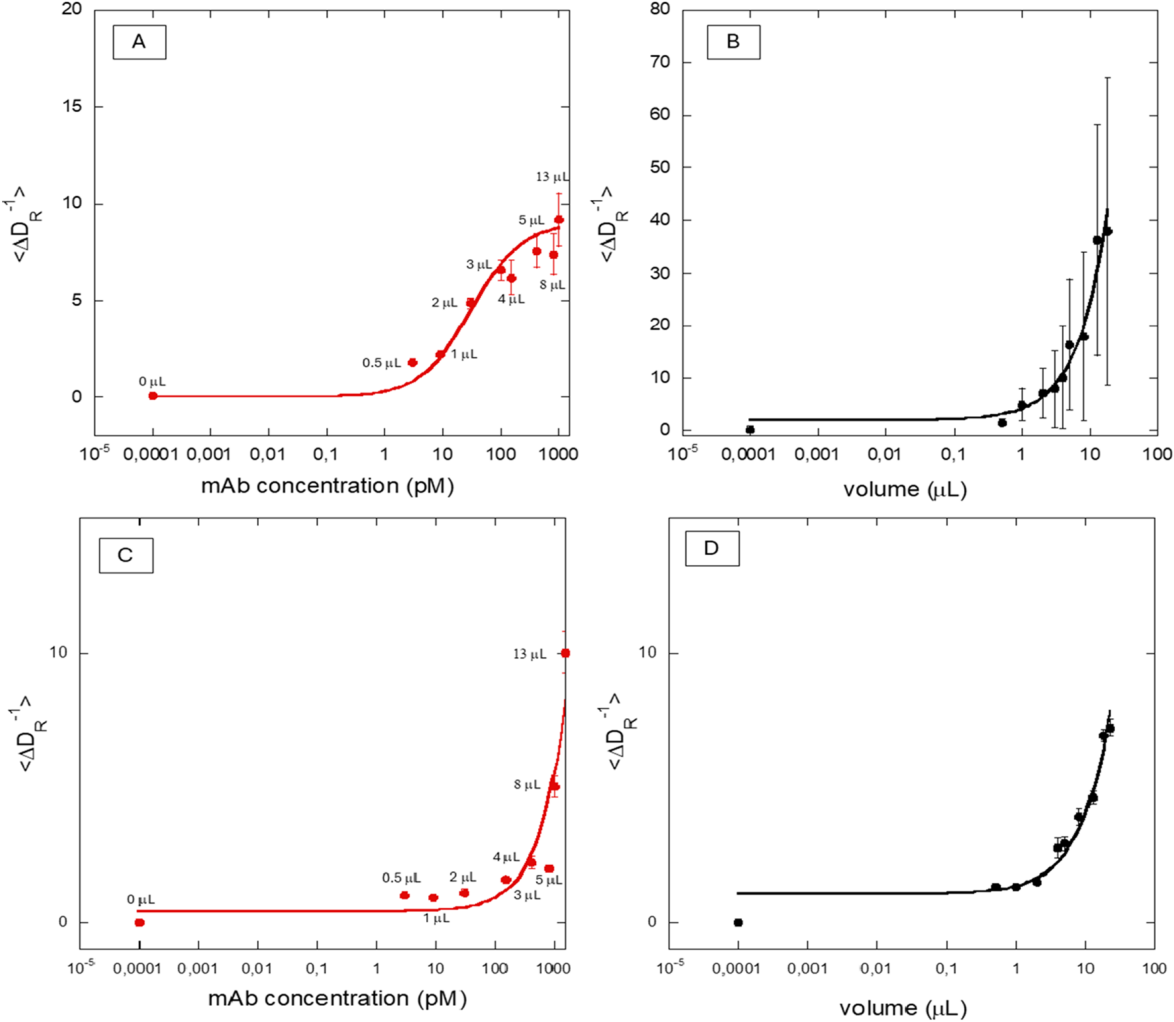
Response of nanobiosensors and GNR + MUA to a spiked sample (positive condition) and to serum matrix (negative condition). (A) Response curve showing the specific binding interaction between monoclonal antibodies (mAb) and the nanobiosensors, exhibiting a sigmoidal profile consistent with binding site saturation. (B) Response curve of nanobiosensors to serum matrix alone, displaying an exponential increase indicative of nonspecific interaction. (C) Positive control, consisting of GNR + MUA, without the immobilized recognition element (N protein), with diluted serum matrix in the presence of the monoclonal antibody (analyte). (D) Negative control, consisting of GNR + MUA, without the immobilized recognition element (N protein), added to the diluted serum matrix alone, without the presence of the monoclonal antibody (analyte).

Figure 4A shows the nanobiosensor response in the presence of an artificially positive sample (serum matrix + antibody), whereas Figure 4B shows the response in the presence of the same volume of serum matrix without antibody addition. Figure 4C,4D present the response of GNRs + MUA (nanobiosensor control) in the presence of the artificially positive sample (serum matrix + antibody) and in the presence of the same volume of serum matrix without antibody addition, respectively.

In Figure 4A, ⟨Δ*D*_R_^–1^⟩ exhibited a sigmoidal dose–response profile, consistent with the bivalent binding behavior of antibodies, which possess two identical antigen-binding sites and may display cooperative binding kinetics. This profile reflects the relationship between antigen concentration and antibody affinity. At higher antigen concentrations ⟨Δ*D*_R_^–1^⟩ reached a plateau near 9 μs, in agreement with the expected hydrodynamic size of the monoclonal antibody fragment employed (≈6 nm, ≈53 kDa). The low standard deviation associated with these mean values indicate signal stabilization within the acquisition window, suggesting that under the experimental conditions adopted, the system had reached a steady-state regime.

In contrast, Figure 4B showed a continuous, nonsaturating increase in ⟨Δ*D*_R_^–1^⟩, indicating that the absence of specific molecular recognition favors nonspecific adsorption and mild aggregation effects at the nanobiosensor surface. Both control responses (Figure 4C,D) also exhibited a monotonic increase in ⟨Δ*D*_R_^–1^⟩, confirming the absence of specific interactions. Overall, Figure 4 clearly distinguishes nonspecific matrix effects from the specific binding mediated by the monoclonal antibody.

### Serological Tests

Serological assays were conducted using a set of ten well-characterized clinical serum samples provided by CTVacinas (for more information, see Supporting Information S2). Each sample was diluted 1:1000 in PBS (pH 7.4) and individually tested by adding 20 μL to wells containing nanobiosensors (NBs) at a final concentration of 130 pM. All experiments were performed in triplicate. Immediately after sample addition, consecutive time-ACFs were recorded for approximately 10 min. For each time point, the mean value ⟨Δ*D*_R_^–1^⟩ = ⟨*D*_RS_^–1^⟩ – ⟨*D*_R0_^–1^⟩ was calculated.

The temporal response curves of ⟨Δ*D*_R_^–1^⟩ for negative and positive serum samples are presented in Figure 5. For negative sera (Figure 5A), two complementary analytical parameters were extracted: (i) the initial slope of the response curve, calculated from the first four data points, and (ii) the saturation value, determined from the last six points. The mean angular coefficient of the initial slope was (0.084 ± 0.010), and saturation occurred near 20 μs. This slight variation in the rotational diffusivity likely reflects a basal response arising from nonspecific interactions between the nanobiosensors and the serum matrix components. For positive sera (Figure 5B), the response curves exhibited a markedly steeper initial slope, with angular coefficients ranging from 0.12 to 0.25, indicating faster association kinetics between NBs and antinucleocapsid (anti-N) immunoglobulins. A correlation is seen between the assay intervals in days that are summarized in Table S1 (Supporting Information) and the initial slope of the positive sera response curves. Values of ⟨Δ*D*_R_^–1^⟩ were consistently higher than those observed in negative samples, reflecting the formation of specific antigen–antibody complexes. Considering the previously observed basal effect, these measured values are consistent with the estimated hydrodynamic size of immunoglobulins (≈12 nm).^35^ For diagnostic discrimination, a cutoff value of (18 ± 6) μs was established, corresponding to the mean negative response plus two standard deviations. Figure 5C illustrates the integration of this threshold, defining a gray zone around the cutoff to account for sample variability, and the characteristic positive response at 300 s. Collectively, these findings demonstrate that the DDLS-based nanobiosensor can discriminate against clinical samples previously classified as positive or negative according to the optical density (OD) values obtained by ELISA test.^29^ The presented method consistently distinguished between the two groups, demonstrating agreement with the classification established by the reference assay (Table S1). These findings indicate that the proposed strategy presents adequate discriminatory capacity, reinforcing its potential application in diagnostic analyses.

**Figure 5:**
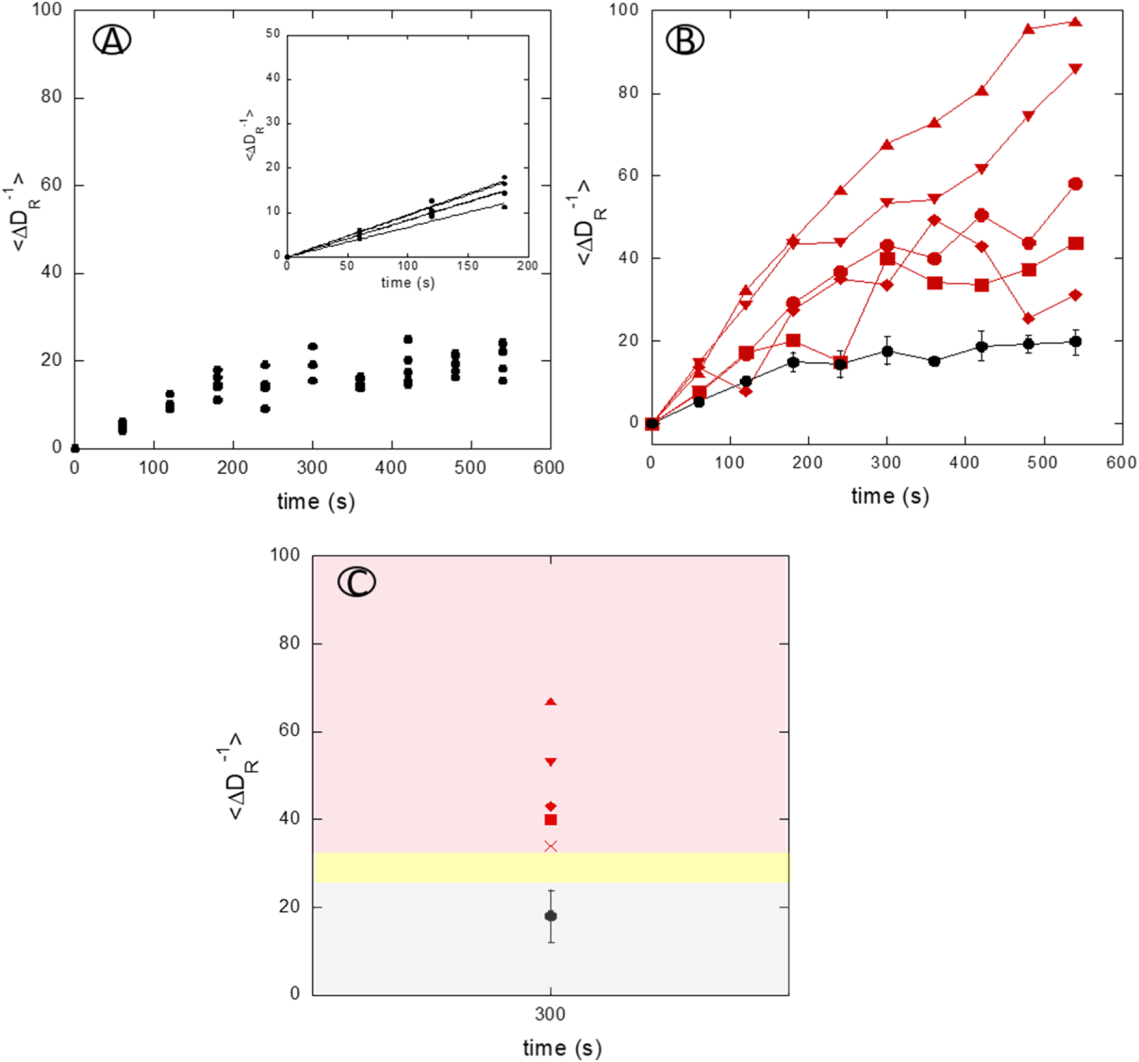
Kinetic response profiles of serum samples obtained by DDLS measurements over time. (A) Negative serum response: serum samples from negative controls (black) showed minimal increases in ⟨*D*_R_^–1^⟩, remaining below 20 μs, consistent with nonspecific background signals. (Inset) The initial slope of the response curve yields an average angular coefficient of (0.084 ± 0.010). (B) Positive serum response: serum samples from positive controls (red) exhibited a progressive increase in ⟨*D*_R_^–1^⟩ over time, indicative of specific binding interactions between nanobiosensors (NB) and immunoglobulins against protein N, in agreement with the expected hydrodynamic size of immunoglobulins (≈12 nm). (C) Final ⟨*D*_R_^–1^⟩values: summary of final ⟨*D*_R_^–1^⟩ values, showing a clear separation between positive (red zone) and negative (gray zone) sample groups. The cutoff value, derived from the distribution of negative samples, enables discrimination between specific and nonspecific responses, with an indeterminate zone (yellow zone) accounting for sample variability near the threshold.

In addition, the results highlight the potential of the proposed platform for future point-of-care testing (POCT) applications. The use of a portable DDLS-based reader combined with bioconjugated gold nanorods enables rapid and label-free detection, which is key for portable diagnostic systems. Furthermore, the short analysis time and low operational complexity of the custom-built reader support its applicability for on-site measurements. Future developments may focus on validation using larger and more diverse sample sets to define diagnostic sensitivity and specificity, aiming to enable practical implementation in real-world diagnostic scenarios.

## Conclusions

This work demonstrates the proof-of-concept of an innovative label-free diagnostic platform based on Dynamic Depolarized Light Scattering (DDLS) analysis of functionalized gold nanorods. By monitoring alterations in the rotational diffusion coefficient (*D*_R_) of nanobiosensors dispersed in solution, the system enables the direct and real-time detection of biomolecular interactions without the need for secondary labeling or amplification steps. The custom-built portable DDLS reader provides rapid measurements (≈10 s) with low operational complexity. Serological assays using diluted clinical samples demonstrated the ability of the methodology to differentiate specific antibody–antigen interactions from nonspecific bindings, highlighting its robustness and applicability under realistic conditions. Although additional validation with a larger sample set and additional target analytes is still required, the proposed DDLS-based nanobiosensing platform shows strong potential for future integration into portable point-of-care testing (POCT) devices. Such developments could enable rapid, on-site diagnostics in complex matrices, contributing to more accessible and efficient diagnostic strategies.

## Supplementary Material



## Notes

Additional figures, experimental details, supporting tables, and any other materials referenced in the manuscript (PDF)
