# Supplementary material for: Label-Free Nanostructured Biosensing Platform Based on Depolarized Dynamic Light Scattering for Rapid and Portable Detection of Immunoglobulins in Complex Biological Samples
Source: ACS Omega. 2026 May 21;11(21):30755–64. doi: 10.1021/acsomega.5c12965 (PMC13234887; doi:10.1021/acsomega.5c12965)
Supplement: Supplementary file 1 [file ao5c12965_si_001.pdf]

# Label-free nanostructured biosensing platform based on depolarized dynamic light scattering for rapid and portable detection of immunoglobulins in complex biological samples

Caroline Magalhães Junqueira,[†,‡,¶] Kennedy Batista Gonçalves,[¶,‡] Lívia de Brito Macedo,[†,‡] Patrick Gonçalves Mendes,[¶,‡] Nick Rocha Costa,[¶,‡] Iara Borges Apolinário,[‡] Enzo Morrison Figueiredo Costa Jorge,[¶,‡] Amanda Bonoto Gonçalves,[§,‡] Anna Carolina Pinheiro Lage,[§] Daniel de Assis Santos,[†] Flávio Guimarães da Fonseca,[†] Rosimeire Coura Barcelos,[ǂ] Ary Corrêa Junior,[†,‡] Luiz Orlando Ladeira,[¶,‡] Oscar Nassif de Mesquita,[¶,‡] and Lívia Siman Gomes[*,¶,‡]

†*Department of Microbiology, Universidade Federal de Minas Gerais (UFMG), Belo Horizonte, MG, Brazil*

‡*Center of Technology in Nanomaterials and Graphene (CTNano), Universidade Federal de Minas Gerais (UFMG), Belo Horizonte, MG, Brazil*

¶*Department of Physics, Universidade Federal de Minas Gerais (UFMG), Belo Horizonte, MG, Brazil*

ǂ*GAD – Organic Chemistry Group, Federal University of São João del-Rei, Divinópolis, 35501-296, Minas Gerais, Brazil*

§*Rene Rachou Institute (Fiocruz Minas), Belo Horizonte, MG, Brazil*

E-mail: liviasg@fisica.ufmg.br

# Supporting Information

# S1. Antigen production

Antigen production was carried out by the Vaccine Technology Center (CTVacinas) at the Federal University of Minas Gerais (UFMG). The full-length coding sequence of the nucleocapsid (N) gene of SARS-CoV-2 (GenBank accession number: MT126808.1) was optimized, subcloned into the pET-24a (+) expression vector, and used to transform *E. coli* BL21(DE3). Plasmid-positive clones were cultured in LB medium, and the expressed protein was purified via affinity chromatography using nickel columns on an AKTA prime plus system, following the manufacturer's protocol (GE Healthcare, USA).

# S2. Clinical samples

Clinical serum samples were provided by the Center for Vaccine Technology (CTVacinas) at the Federal University of Minas Gerais (UFMG). Negative sera, collected prior to 2020, were obtained from healthy donors with no known history of SARS-CoV-2 exposure. Additional negative sera were sourced from individuals who tested negative for SARS-CoV-2 via qRT- PCR of nasal swabs. Positive samples were selected based on a documented history of a positive SARS-CoV-2 PCR result from nasal swabs or confirmed by the rapid dual-path platform (DPP) COVID-19 IgM/IgG test (Bio-Manguinhos, FioCruz, Brazil).

Table S1 presents the comparison between PCR and ELISA results obtained

for the serum samples analyzed in this study. Samples 1 to 5 were confirmed as positive by PCR, showing different optical density (OD) values of ELISA. In contrast, samples 6–10 were PCR-negative, exhibiting absorbance levels near the background signal. The interval between assays is also provided.

**Table S1.** Comparison between PCR and ELISA results, and assay interval for serum samples.

| **Serum sample** | **1** | **2** | **3** | **4** | **5** | **6** | **7** | **8** | **9** | **10** |
|---|---|---|---|---|---|---|---|---|---|---|
| PCR result | Pos | Pos | Pos | Pos | Pos | Neg | Neg | Neg | Neg | Neg |
| ELISA (absorbance) | 7.2 | 1.5 | 5.5 | 3.0 | 1.8 | 0.4 | 0.8 | 0.3 | 0.5 | 0.3 |
| Assays interval [days] | 13 | 20 | 30 | 18 | 26 | ND | ND | ND | ND | ND |

*Note:* POS: positive; NEG: negative; ELISA optical density (OD) values were interpreted according to the assay cutoff: OD < 0.6 = low negative, 0.6–0.8 = high negative, 0.8-1.1= borderline; 1.1-2.1= low positive and > 2.1 = high positive. ND= no data.

# S3. DDLS Theory

In a typical DDLS experiment, the normalized intensity time ACF, or $g_{vh}^2$ ($t$), of the depolarized scattered light is measured. This function can be expressed as an integral of the product of intensities (I) at time t and delayed time (t + $t_d$):

$$ACF = g_{vh}^2(t) = \frac{\langle I(t)I(t+\ t_d)\rangle}{\langle I(t)\rangle^2} \qquad \text{(S1)}$$

In the proposed methodology, $t_d$ is set as an integer multiple of the camera time bin, which corresponds to approximately 23 $\mu$s (1/42533Hz).

For monodisperse particles that undergo Brownian motion, $g^2$ ($t$) decays exponentially with an exponential time constant $\tau$ related to a decay rate $\Gamma$ ($\tau = \Gamma^{-1}$):

$$g_{vh}^2(t) = \beta\, e^{-2\Gamma t} + 1 \quad \text{(S2)}$$

$\Gamma$ is related to both translational $\Gamma_T$ and rotational $\Gamma_R$ rates:

$$\Gamma = \Gamma_T + \Gamma_R = D_T\, q^2 + 6D_R \quad \text{(S3)}$$

where $\beta$ is a coherence factor (typically 1) and the magnitude of the wave vector $q$ is given by $q = \frac{4\pi}{\lambda} n \sin\left(\frac{\theta}{2}\right)$, $n$ is the refractive index of the solution, $\lambda$ is the wavelength of the incident light, and $\theta$ is the scattering angle between the incident and scattered light directions. $D_T$ and $D_R$ are the translational and rotational diffusion coefficients, respectively. In this work, the decay of the depolarized intensity autocorrelation function is interpreted primarily in terms of rotational diffusion. To quantitatively support this approximation, we explicitly compare the magnitudes of the rotational and translational contributions under the experimental conditions employed ($\theta$ = 173$\circ$ , $\lambda$= 650 nm, q $\approx 2.57 \times 10^7$ m$^{-1}$). For our typical nanobiosensor (L = 47 nm, D = 22 nm), the decay rates calculated are $\Gamma_R \approx$ 147 kHz and $\Gamma_{tr} \approx$ 10.6 kHz, giving a ratio $\Gamma_T/\Gamma_R \approx 0.072$. In this regime, the condition $\Gamma_T/\Gamma_R \ll 1$:

$$(qL)^2 \ll 54\frac{G(AR)}{F(AR)} \quad \text{(S4)}$$

is well satisfied, justifying the approximation $\Gamma \simeq 6D_R$.

After biorecognition with antibodies, the effective hydrodynamic dimensions approximately increase to L = 69 nm and D = 32 nm. The corresponding decay rates become $\Gamma_R \approx$ 47.2 kHz and $\Gamma_T \approx$ 7.29 kHz, resulting in $\Gamma_T/\Gamma_R \approx 0.152$. Although the relative contribution of translational diffusion increases, the inequality above remains fulfilled, and rotational diffusion continues to dominate decay.

# S.4 ACF Fitting model

The use of a monoexponential fitting model is justified from an experimental standpoint. Measurements are performed using a camera with a high acquisition rate, such that the detection window for a complete ACF is approximately 23 ms. Under these conditions, the ACFs obtained for gold nanorods and the nanobiosensor in the presence of sample exhibit well-behaved decays that can be adequately described by a single exponential, with low fitting residuals (Figure S1).

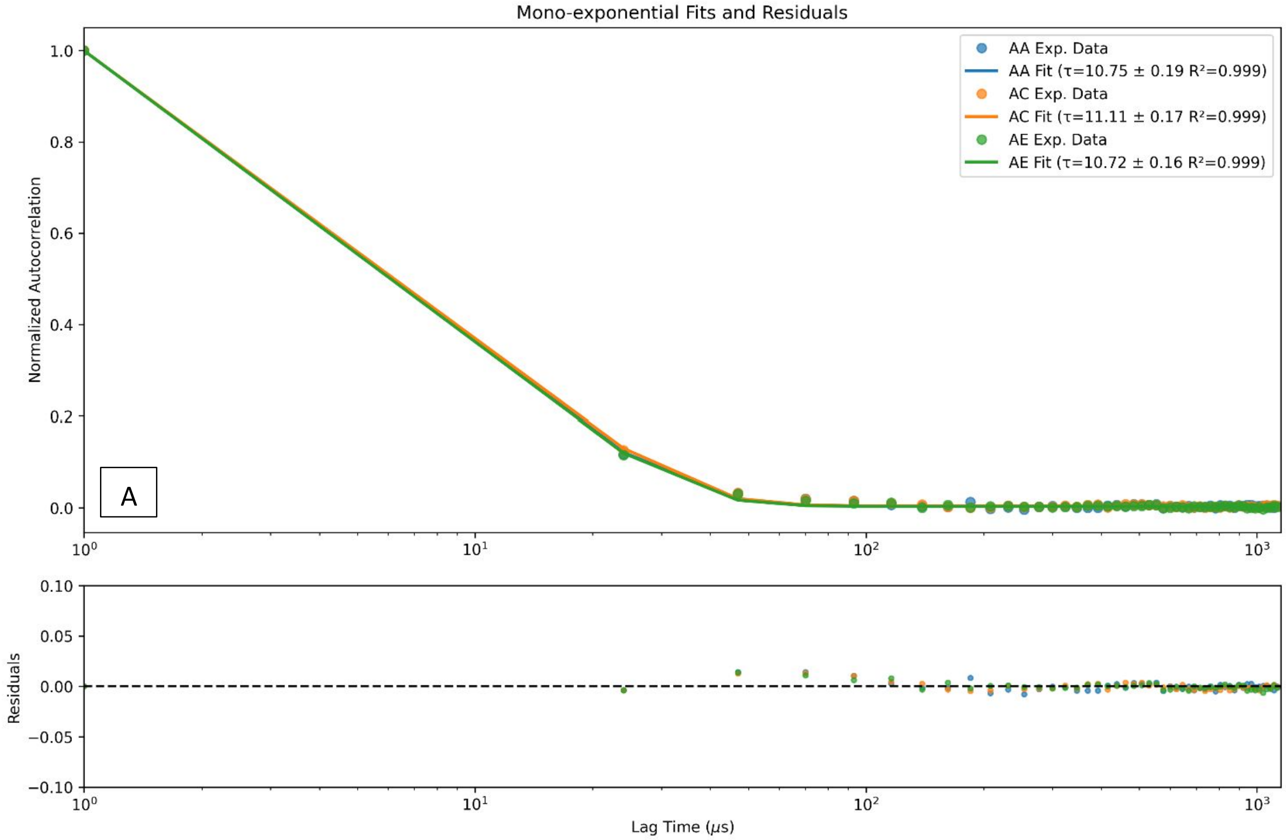
Mono-exponential Fits and Residuals
AA Exp. Data
AA Fit (τ=10.75 ± 0.19 R²=0.999)
AC Exp. Data
AC Fit (τ=11.11 ± 0.17 R²=0.999)
AE Exp. Data
AE Fit (τ=10.72 ± 0.16 R²=0.999)
Normalized Autocorrelation
A
Residuals
Lag Time (μs)

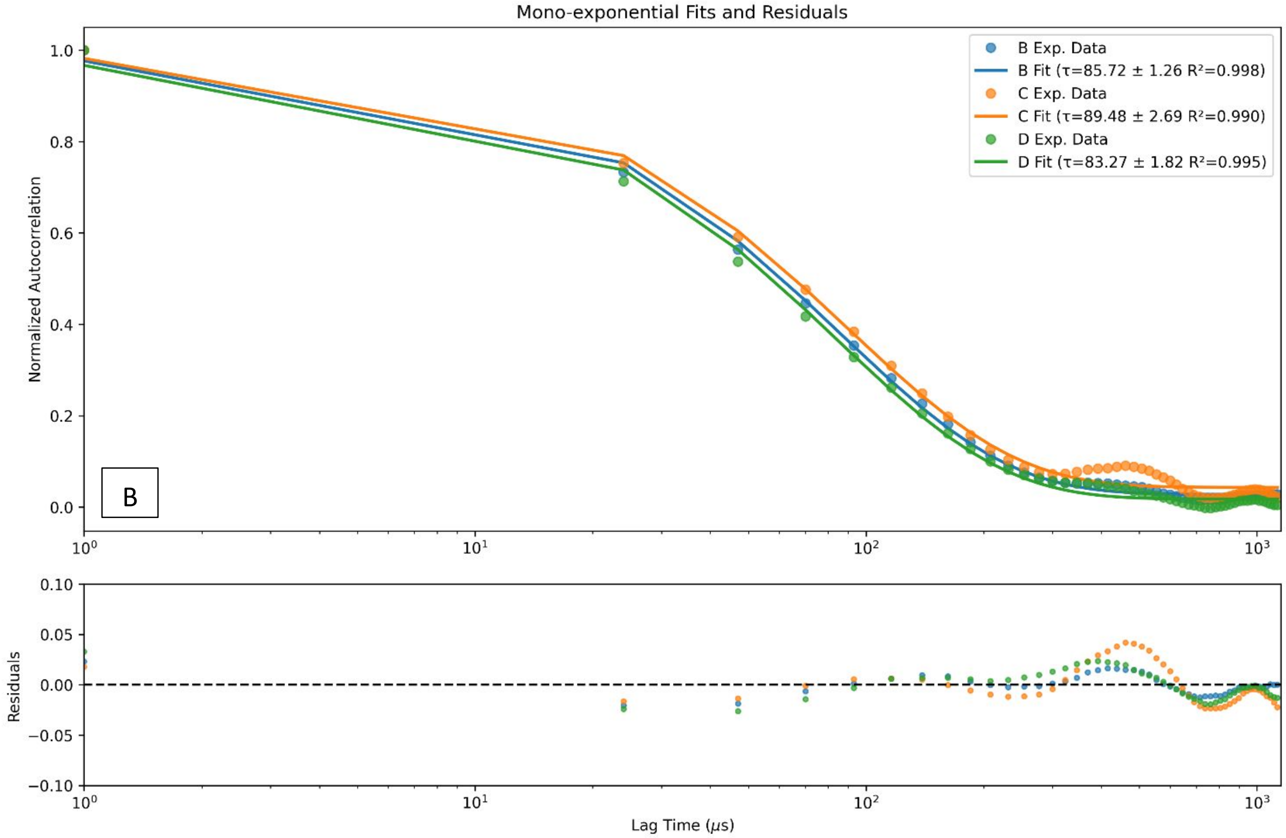
Mono-exponential Fits and Residuals
B Exp. Data
B Fit (τ=85.72 ± 1.26 R²=0.998)
C Exp. Data
C Fit (τ=89.48 ± 2.69 R²=0.990)
D Exp. Data
D Fit (τ=83.27 ± 1.82 R²=0.995)
Normalized Autocorrelation
B
Residuals
Lag Time (μs)

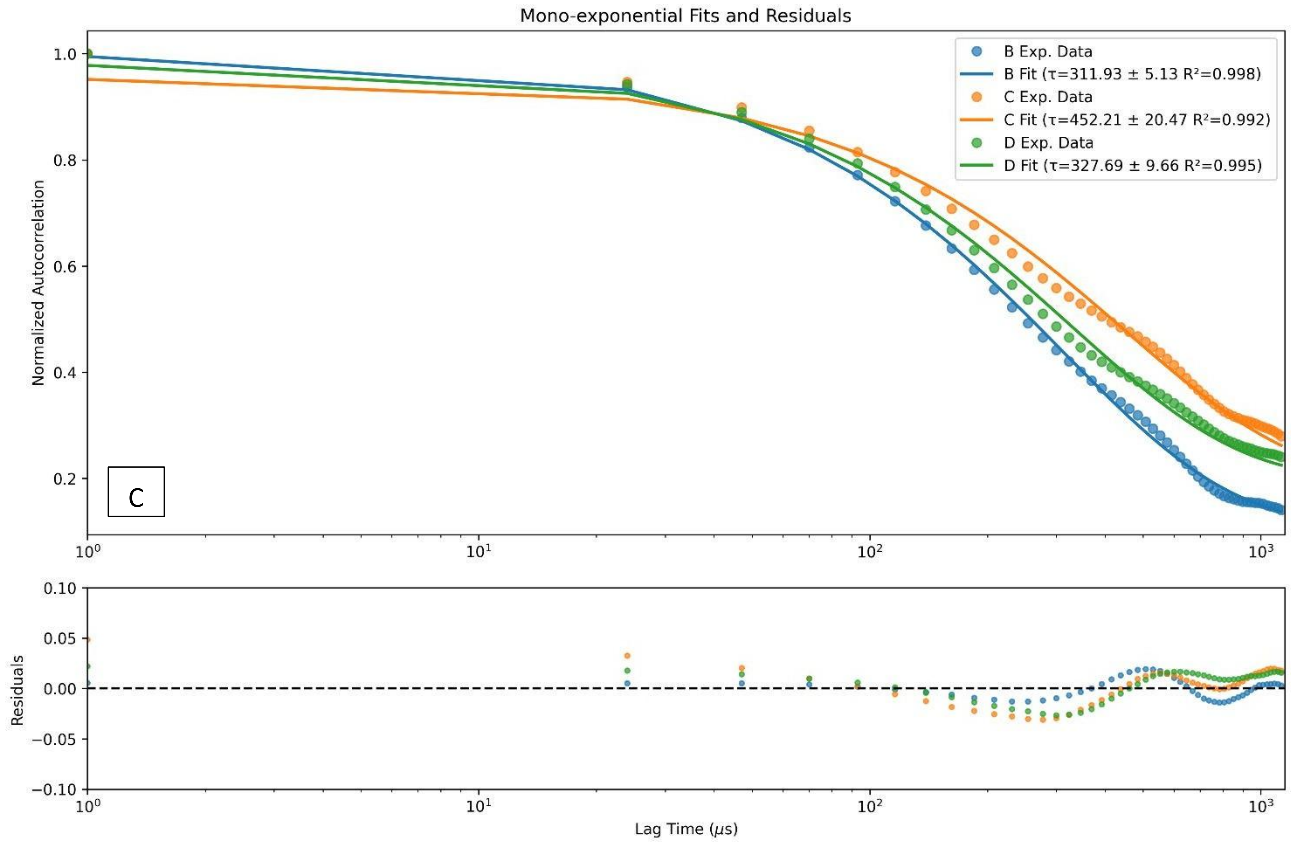


**Figure S1.** ACFs fitting model. Top: Normalized ACFs of three different correlations of: (A) gold nanorods and the nanobiosensor in the presence of (B) negative and (C) positive samples. Bottom: Residuals of the fits.

Additionally, the characteristic decay times (τ) extracted from multiple measurements (≈ 30 measurements) show a narrow distribution with low dispersion (Figure S2). Altogether, those results indicate that, under the experimental conditions employed, there is no significant evidence of dominant contributions from rare slow-rotating species, such as aggregates or transient dimers.

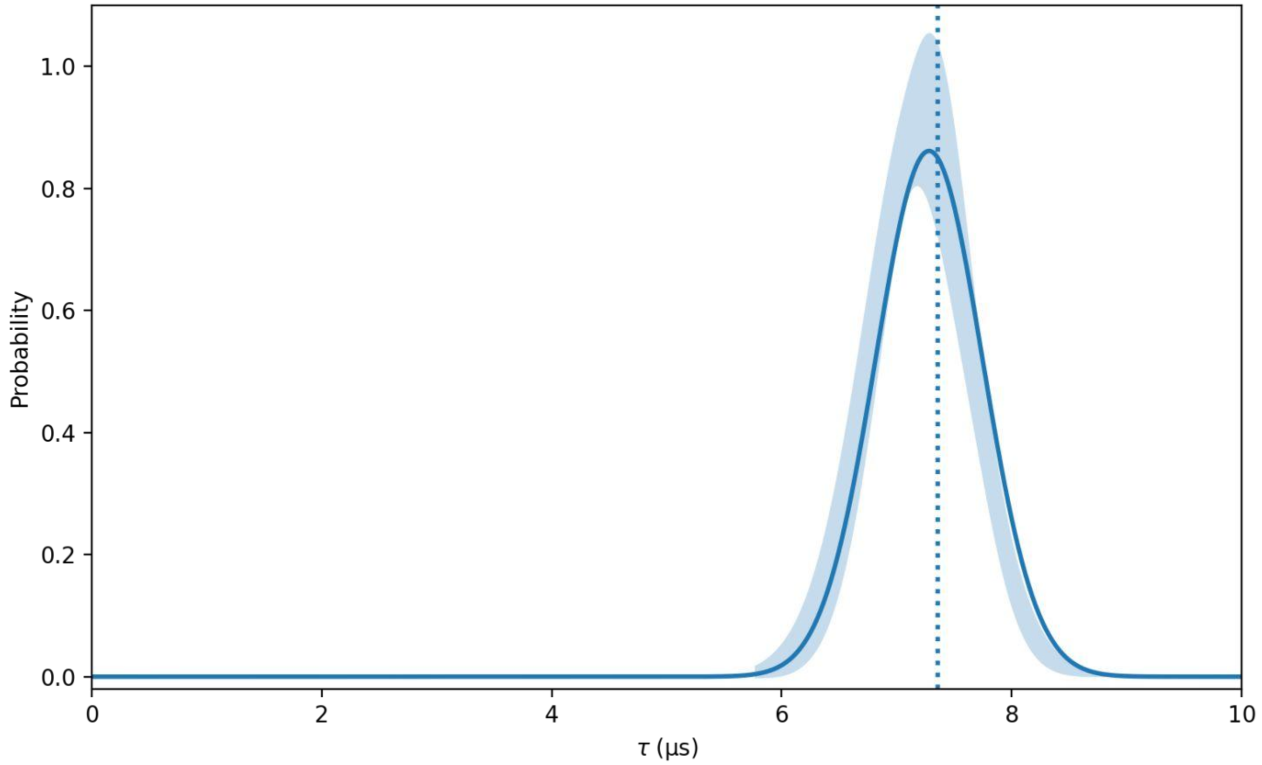


**Figure S2.** Distribution of the decay time (τ, μs) fitted with a Gaussian model. Normalized distributions of the ACFs decay time. The line represents the estimated probability density function, while the shaded area indicates the dispersion (± standard deviation) around the mean. The dashed vertical line corresponds to the mean τ value $<\tau> = (7.3 \pm 0.5)$ μs.

# S.5 Theoretical Description of GNRs Diffusivity

The rodlike, cylindrical shape is frequently found in colloidal particles, and their hydrodynamic properties in a dilute solution have been the subject of much theoretical and computational work. As suggested by de la Torre et al., the theoretical expressions for translational and rotational diffusion of straight cylinders of length L, cross-sectional diameter d and aspect ratio AR = L/d are of the form:

$$D_T = \frac{k_B T}{3\pi\eta L} F(AR) \tag{S6}$$

$$D_R = \frac{3k_B T}{\pi\eta L^3} G(AR) \tag{S7}$$

where $k_B$ is the Boltzmann constant, T is the absolute temperature, n is the viscosity of the liquid and L denotes the length of the rod. F(AR) and G(AR) are model-dependent functions and called “end-effect” corrections of the aspect ratio. For straight cylinders:

$$F(AR) = \ln(AR) + 0.312 + \frac{0.565}{AR} - \frac{0.1}{AR^2} \quad \text{(S8)}$$

$$G(AR) = \ln(AR) - 0.662 + \frac{0.917}{AR} - \frac{0.05}{AR^2} \quad \text{(S9)}$$

The equations for F(AR) and G(AR) cover AR = 0.1 to 20. Considering equations S7 and S9 an approximate equation for the length L can be derived:

$$L = \left[\frac{3k_BT}{\pi\eta D_R} G(AR)\right]^{\frac{1}{3}} \quad \text{(S10)}$$

# S6. Characterization of gold nanorods

**UV-Vis measurements**

UV-Vis absorption spectra were recorded in a multiwell plate format. Each sample consisted of 350 $\mu$L, including gold nanorods (50 pM), GNR functionalized with MUA (200 pM), and the final nanobiosensors (200 pM). Measurements were performed at room temperature using a Thermo Scientific Varioskan LUX spectrophotometer, covering the wavelength range of 400 to 900 nm.

## Zeta potential measurements

Zeta potential was determined using 1 mL of each sample. The solutions analyzed included gold nanorods (50 pM), GNRs functionalized with MUA (10 pM) and nanobiosensors (10 pM). Measurements were performed in triplicate and are reported as mean ± standard deviation. All samples were dispersed in ultrapure water at room temperature (25 °C) and analyzed using a Litesizer 500 (Anton Paar, Austria). The observed changes in the Zeta potential after each functionalization step confirmed the modification of the nanoparticle surface chemistry.

## TEM measurements

Transmission Electron Microscopy (TEM) was employed as the reference technique to determine the accuracy parameter of the optical hardware. For this characterization, 3.5 nM gold nanorod (GNR) and nanoblocker solutions were concentrated by centrifugation (MULTIFUGE X3R, Thermo Scientific, USA) at 10,000 g for 10 minutes. The supernatant was discarded and the precipitate was resuspended in 100 $\mu$L of 3 mM aqueous CTAB solution. The concentrated

samples were sent to the Microscopy Center of UFMG for TEM imaging using a Tecnai G2-12 Spirit Biotwin FEI microscope. Representative GNR TEM images (Fig. S3A) revealed relatively homogeneous morphology, with an average length of (47.2 ± 6.1) nm and a diameter of (24.7 ± 1.8) nm, resulting in a low aspect ratio of (1.9 ± 0.3) (n = 161).

Particle counting and dimensional analysis were performed with a custom MATLAB algorithm based on the ibinarize function. TEM images (Fig. S3B) were processed using gray-level thresholding to obtain binary images, where the particles were identified as connected regions with a value of 1. Each particle was fitted to an ellipse to extract the major axes (L) and the minor axes (d), and the data were stored in CSV format. Statistical analyses of length (L), diameter (d), and aspect ratio (AR) were then performed.

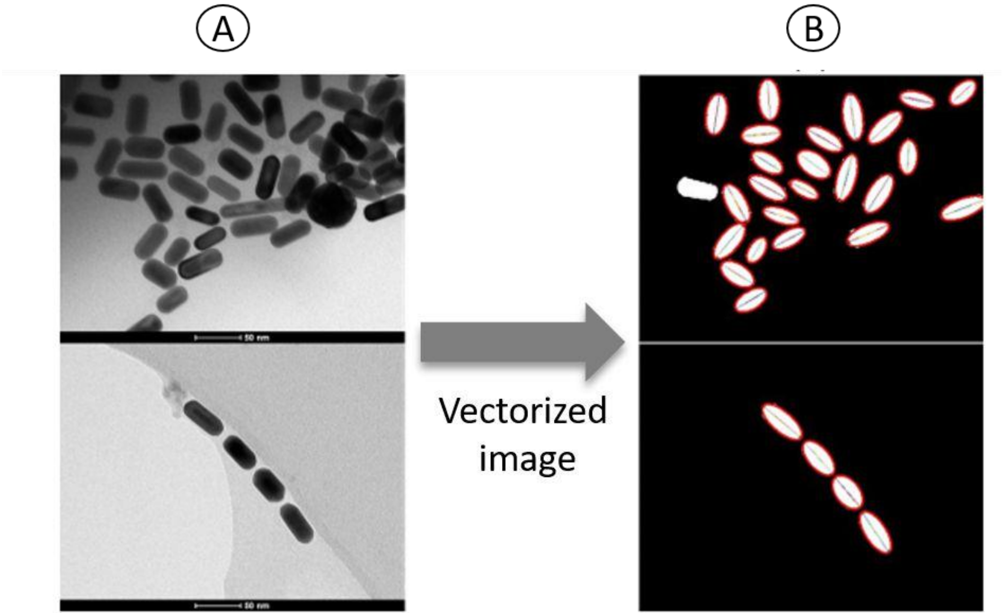


**Figure S3**. Representative GNR TEM images (A) Transmission electron microscopy (TEM) image of gold nanorods (GNRs). (B) The morphology, length, and aspect ratio were determined from the micrographs using an image-processing algorithm developed in MATLAB.

Table S2 summarizes the mean dimensions of the synthesized gold nanorods (GNRs) determined by transmission electron microscopy (TEM) image analysis. The particles exhibited an average length of approximately 47 nm and a diameter of 25 nm, resulting in an aspect ratio close to 2. These morphological parameters confirm the successful synthesis of uniform nanorods suitable for optical biosensing applications.

**Table S2.** Mean dimensions of gold nanorods (GNRs) determined by TEM image analysis.

| Sample | Length (nm) | Diameter (nm) | Aspect ratio (AR) | Particles analyzed |
|---|---|---|---|---|
| GNRs | 47.2 ± 6.1 | 24.7 ± 1.8 | 1.9 ± 0.3 | 161 |

*Note:* The GNRs exhibited an average length of ≈ 47 nm and diameter of ≈ 25 nm, corresponding to an aspect ratio of ≈ 2, confirming uniform morphology suitable for optical biosensing application.

# S7. Evaluation of the nanobiosensor´s sensitivity and selectivity using monoclonal antibody

The sensitivity and selectivity of the DDLS-based biosensor were evaluated using monoclonal antibodies (mAbs) specific to the SARS-CoV-2 nucleocapsid (N) protein. For this, mAbs were spiked into a human serum matrix to generate artificially positive samples. Wells containing NB at 130 pM were titrated with sample volumes corresponding to mAb concentrations ranging from 0.6 pM (0.03 ng/mL) to 800 pM (42.4 ng/mL), hereafter referred to as a positive condition. The effect of serum matrix alone, without added antibodies, was evaluated as a negative condition. Following each sample addition, 60 consecutive time-ACF measurements were recorded. An average value $\langle \Delta D_R^{-1} \rangle$ was calculated for every 10 consecutive measurements and subtracted from the initial $\langle \Delta D_R^{-1} \rangle$, generating $\langle \Delta D_R^{-1} \rangle$ points shown in Figure S4. Figures S5A and S5B show the evolution of $\langle \Delta D_R^{-1} \rangle$ for positive and negative conditions, respectively. For the positive condition, $\langle \Delta D_R^{-1} \rangle$ exhibits a sigmoidal profile, consistent with the bivalent nature of antibodies and the resulting complex binding kinetics. This standard dose-response curve reflects both the antigen concentration and the antibody affinity.  At high antigen concentrations, $\langle \Delta D_R^{-1} \rangle$ reaches a plateau around 9 $\mu$s, which is consistent with the estimated globular size of the mAb (≈ 6 nm). For the negative condition, $\langle \Delta D_R^{-1} \rangle$ increases continuously, indicating that the absence of specific interactions favors aggregation due to nonspecific binding to NB.

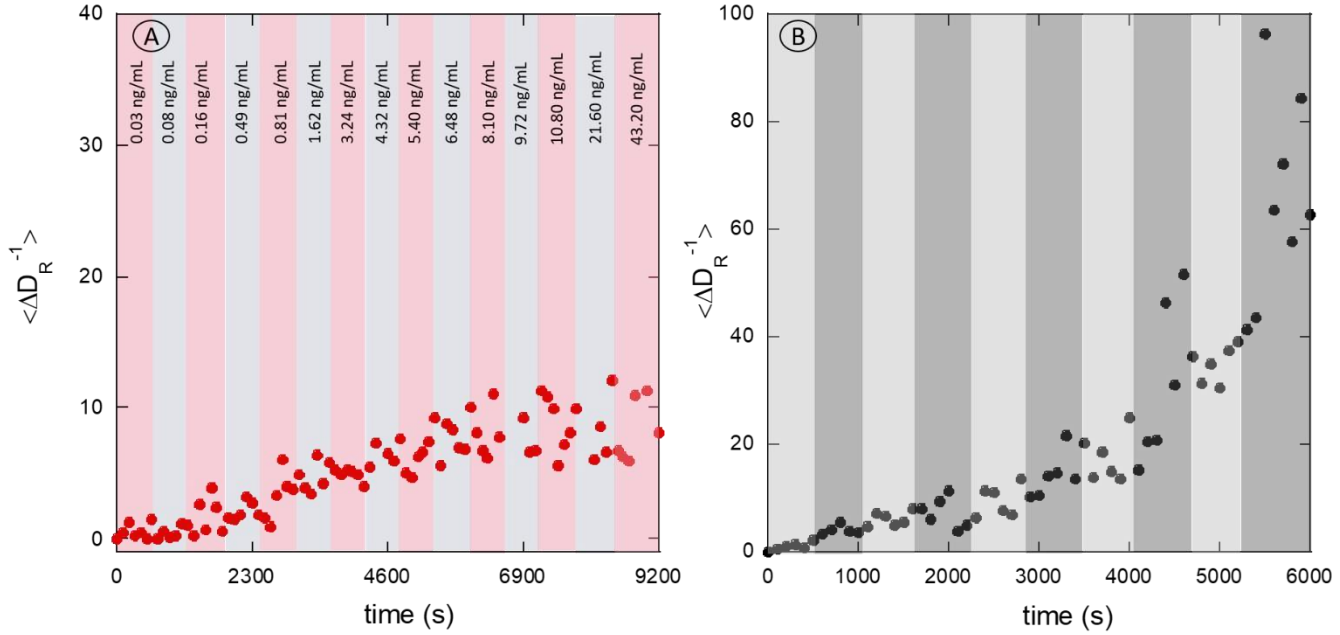


**Figure S4.** Time-dependent evolution of $\langle \Delta D_R^{-1} \rangle$ obtained from kinetic DDLS measurements. (A) Positive condition (red), exhibiting a sigmoidal profile that stabilizes around 10 $\mu$s. These results highlight distinct kinetic behaviors between positive and negative conditions, reinforcing the importance of temporal analysis to discriminate specifically from nonspecific interactions (matrix effects). (B) Negative condition (black), showing a progressive exponential-like increase reaching values above 90 $\mu$s, consistent with cumulative nonspecific matrix effects.
